# Prion-Seeding Activity in Cerebrospinal Fluid of Deer with Chronic Wasting Disease

**DOI:** 10.1371/journal.pone.0081488

**Published:** 2013-11-25

**Authors:** Nicholas J. Haley, Alexandra Van de Motter, Scott Carver, Davin Henderson, Kristen Davenport, Davis M. Seelig, Candace Mathiason, Edward Hoover

**Affiliations:** 1 Department of Microbiology, Immunology, and Pathology, College of Veterinary Medicine and Biomedical Sciences, Colorado State University, Fort Collins, Colorado, United States of America; 2 School of Zoology, University of Tasmania, Hobart, Tasmania, Australia; 3 Department of Veterinary Clinical Sciences, University of Minnesota, St. Paul, Minnesota, United States of America; Rocky Mountain Laboratories, NIAID, NIH, United States of America

## Abstract

Transmissible spongiform encephalopathies (TSEs), or prion diseases, are a uniformly fatal family of neurodegenerative diseases in mammals that includes chronic wasting disease (CWD) of cervids. The early and ante-mortem identification of TSE-infected individuals using conventional western blotting or immunohistochemistry (IHC) has proven difficult, as the levels of infectious prions in readily obtainable samples, including blood and bodily fluids, are typically beyond the limits of detection. The development of amplification-based seeding assays has been instrumental in the detection of low levels of infectious prions in clinical samples. In the present study, we evaluated the cerebrospinal fluid (CSF) of CWD-exposed (n=44) and naïve (n=4) deer (n=48 total) for CWD prions (PrP^d^) using two amplification assays: serial protein misfolding cyclic amplification with polytetrafluoroethylene beads (sPMCAb) and real-time quaking induced conversion (RT-QuIC) employing a truncated Syrian hamster recombinant protein substrate. Samples were evaluated blindly in parallel with appropriate positive and negative controls. Results from amplification assays were compared to one another and to obex immunohistochemistry, and were correlated to available clinical histories including CWD inoculum source (e.g. saliva, blood), genotype, survival period, and duration of clinical signs. We found that both sPMCAb and RT-QuIC were capable of amplifying CWD prions from cervid CSF, and results correlated well with one another. Prion seeding activity in either assay was observed in approximately 50% of deer with PrP^d^ detected by IHC in the obex region of the brain. Important predictors of amplification included duration of clinical signs and time of first tonsil biopsy positive results, and ultimately the levels of PrP^d^ identified in the obex by IHC. Based on our findings, we expect that both sPMCAb and RT-QuIC may prove to be useful detection assays for the detection of prions in CSF.

## Introduction

Chronic wasting disease (CWD) is an efficiently transmitted prion disease of cervids (e.g. deer, elk, and moose), and the only known prion disease affecting free-ranging, non-domestic animals. While the origins of CWD are uncertain, the disease has been present in wild cervid populations of northern Colorado and southern Wyoming for over 40 years [1,2] and has now been identified in both captive and free-ranging cervids in 22 states, 2 Canadian provinces, and the Republic of Korea [3]. With intensified national surveillance efforts, CWD continues to be identified in areas previously thought to be free of infection, including recent discoveries in Iowa, Texas, and Pennsylvania in 2012 [4–6]. The prevalence of CWD varies across North America, but can be as high as 30% in some areas of Colorado and up to 100% in captive populations [7,8]. 

Identification of infected cervids requires a sensitive and specific detection assay. Immunohistochemistry (IHC) of designated target tissues is currently considered the gold standard diagnostic test for CWD and other prion diseases of animals and man. The true sensitivity and specificity of IHC in the detection of infected individuals is unknown, though it has been demonstrated that the assay may underestimate the level of abnormal pathogenic prion protein (PrP^d^) in a given sample due to the necessity of a proteolytic pre-treatment step to abolish cellular prion protein (PrP^C^) cross-reactivity [9–11]. More recently, reports have focused on *in vitro* PrP conversion assays that involve either amplification of the protease-resistant prion protein (e.g. serial protein misfolding cyclic amplification - sPMCA) [10,12], fluorometric quantitation of recombinant PrP seeding activity as measured by thioflavin T binding (e.g. real time quaking-induced conversion – RT-QuIC) [13], or detection of altered PrP conformation (e.g. the conformation-dependent immunoassay - CDI) [14,15]. 

Definitive identification of infected individuals also relies on selection of an appropriate diagnostic specimen [16–18]. In cervids, the retropharyngeal lymph nodes have been shown to identify infected animals at earlier stages of disease [16,18], though in other species, including cattle and man, the obex (a region of the caudal medulla) is generally considered to be the tissue of choice for the detection of TSE infection [19]. As retropharyngeal lymph node and obex samples are only available postmortem, efforts over the past decade have been made to identify peripherally accessible samples from which an antemortem diagnosis may be made. To this end, several investigators have examined peripheral lymphoid tissues of sheep and deer (e.g. tonsil and recto-anal mucosal associated lymphoid tissue, RAMALT) for antemortem detection of prion infection and found sensitivities (when compared to obex IHC) approaching 100% in both pre-clinical and clinical animals [20–25]. More recently, using cerebrospinal fluid (CSF), amplification or fluorometric seeding activity assays have shown promise in distinguishing TSE-infected and uninfected individuals, including CWD-infected elk [26], scrapie-infected hamsters [27], and humans with Creutzfeldt-Jakob disease [28,29]. 

In the present study, we have applied a standardized sPMCA incorporating polytetrafluoroethylene (PTFE, Teflon®) beads (i.e. sPMCAb) [30] and RT-QuIC methodologies to examine the CSF of 48 CWD-exposed and naïve white-tailed deer (*Odocoileus virginianus*) with known inoculum source (e.g. blood, saliva, brain, etc.), genotype, survival periods, and serial tonsil biopsy IHC results. We hypothesized that sPMCAb and RT-QuIC results would correlate positively with IHC results, as well as to one another, suggesting CSF amplification may be a reliable means of antemortem detection of prion infection. We also sought to identify which specific historical correlates (e.g. incubation periods, etc.) were the strongest predictors of assay results derived from sPMCAb and RT-QuIC. We hypothesized that deer showing protracted signs of CWD, based on longitudinal clinical and lymphoid biopsy history, were more likely to show positive CSF amplification in either assay.

## Materials and Methods

### Ethics Statement

 All animals were inoculated and maintained in dedicated, restricted access, indoor CWD research facilities in accord with Colorado State University Institutional Animal Care and Use Committee guidelines.  The animal care and use committee has approved this study under ACUC approval number 08-175A-01.

### Infected cervids

 In previous studies, forty eight white-tailed deer (*Odocoileus virginianus*) were exposed to CWD from positive or negative sources in various forms (e.g. urine and feces, saliva, environmental fomites, blood or blood components, or brain tissue) and by various routes (e.g. orally, intravenously, intracranially, or through environmental exposure) [31–33]. The sources of inoculum included terminally-ill white-tailed or mule deer (*Odocoileus hemionus*) of unknown PrP genotype (courtesy of Michael Miller, Colorado Division of Parks and Wildlife; Terry Spraker, Colorado State University; the National Park Service; and the Wisconsin Department of Natural Resources), and sub-passage studies in white-tailed deer of either of two genotypes: homozygous for glycine (i.e. G/G) at cervid Prnp position 96, or heterozygous at that position, with alleles for glycine and serine (G/S). Study deer (summarized in Table **1** and Table **S1**) were selected on the basis of availability of CSF for analysis. Cerebrospinal fluid samples were collected cleanly early in the postmortem examination through aspiration from the ventral subarachnoid space at the atlanto-occipital site and frozen at -80°C for a range of 2-5 years prior to analysis.

**Table 1:** Summary of background information and sPMCAb, RT-QuIC, and IHC results from individual deer.

**Animal ID**	**Inoculum**	**Initial tonsil + date (months)**	**Duration of Clinical Signs (weeks)**	**Mean IHC Score**	**Mean PMCA Score**	**Mean RT-QuIC Score**
106	Positive brain	12	3	1	1	0.52
107	Saliva	NA	0	0	0	0
108	Whole blood	12	16	0	0.5	0
110	Whole blood	12	28	1	1	0.67
111	Urine/feces	NA	0	0	0	0
114	Whole blood	19	0	0	0	0.2
122	Saliva	12	0	0.33333	0	0
123	Negative brain	NA	0	0	0.17	0
124	Urine/feces	NA	0	0	0	0
132	Saliva	19	0	0.66667	0	0
133	Whole blood	12	14	1	1	0.38
134	Urine/feces	NA	0	0	0	0
136	Positive brain	12	52	1	1	0.38
137	Whole blood	19	0	0.16667	1	0
138	Brain	6	52	1	1	0.85
141	Urine/feces	NA	0	0	0	0
143	Positive brain	12	4	0.33333	0	0
144	Saliva	12	0	1	1	0.28
147	Saliva	NA	0	0	0	0
150	Urine/feces	NA	0	0	0	0
302	Whole blood	12	9	0.83333	0	0
303	B cells	12	0	0.66667	0	0
307	Monocytes	NA	0	0	0	0
311	Monocytes	NA	0	0	0	0
322	B cells	19	0	0.83333	0	0
324	Platelets	19	0	0	0	0
346	B cells	12	0	1	0	0
347	Whole blood	6	9	0.33333	0	0
357	Platelets	19	0	0	0	0
360	Monocytes	NA	0	0	0	0
371	B cells	19	0	0	0	0
372	Whole blood	12	8	1	1	0.22
373	Monocytes	NA	0	0	0	0
374	Whole blood	6	8	1	0	0
4116	Whole blood	12	0	1	1	0.52
4117	Plasma	NA	0	0	0	0
4119	Whole blood	12	10	0.5	1	0.3
4128	Blood cells	12	0	1	1	0.24
4129	Environment	19	0	0.16667	0.5	0
4153	Plasma	NA	0	0	0	0
4461	Environment	19	0	1	0	0
4488	Negative brain	NA	0	0	0	0
4502	Whole blood	6	12	1	0.5	0.57
4506	Blood cells	19	0	1	0	0.38
4509	Negative brain	NA	0	0	0	0
4513	Whole blood	6	2	1	1	1
4516	Negative brain	NA	0	0	0	0
4521	Blood cells	19	0	0.16667	0	0

Data on the inoculum, earliest tonsil biopsy positive result (in months), and duration of clinical signs (in weeks) prior to necropsy were collected on experimentally inoculated deer. Scores from each experimental assay were normalized and correlated to clinical histories.

### Preparation of normal brain homogenate for sPMCA

 To reduce the risk of contamination, normal brain homogenate (NBH), the substrate for prion conversion *in vitro* by sPMCA, was prepared from *Tg*(*CerPrP*)*5037* mice in a room that had not previously been used for prion research [10,34–36]. Following euthanasia and perfusion with 5mM EDTA in phosphate-buffered saline (PBS), whole brain was collected from naïve *Tg*(*CerPrP*)*5037* mice and placed on ice. Brain homogenates were prepared as a 10% (w/v) solution in PMCA buffer (1% triton-X 100 [v/v], 5mM EDTA, and 150mM NaCl in PBS adjusted to a pH of 7.2) with the addition of Complete Protease Inhibitors (Roche Pharmaceuticals, Indianapolis, IN) using a dounce homogenizer. Homogenates were then centrifuged for 1 minute at 2000rpm and the supernatant frozen in single-experiment aliquots at –80°C in a room in which neither prion containing or potentially exposed materials or equipment were ever present until use in sPMCA. Each preparation was composed of brain from 4-6 mice to minimize the potential influence of expression variation in CerPrP or other co-factors [37,38]; multiple preparations of NBH were utilized over the course of the experiments. 

### Serial PMCA of cerebrospinal fluid

 Cerebrospinal fluid was initially coded to allow for blinded evaluation by sPMCA, then thawed briefly with 10μl of test or control CSF added to 50μl of NBH with the addition of three 1/16” polytetrafluoroethylene (Teflon®) beads (i.e. sPMCAb, McMaster-Carr). Each test sample was evaluated in duplicate and in parallel with positive and negative controls, in adjacent wells of a 96-well plate (USA Scientific, Ocala, FL); along with NBH prepared from unexposed *Tg*(*CerPrP*)*5037* mice as additional, unseeded negative controls. In each experimental run, between 25-35% of samples evaluated were from sham-inoculated deer (n=56/171 total replicates), and an additional 10-20% (n=25/171 total replicates) of the samples were unspiked, NBH negative controls. After sample loading, plates were sonicated using an ultrasonic processor (Qsonica, Newtown, CT) and incubated at 37°C. Sonication parameters entailed 40 second bursts at power level 7.0, followed by 30 minutes of incubation. Each duplicate sample was processed through 92 cycles of sonication performed over 48 hours, followed by a transfer of a 10µl aliquot of sonicated material into 50μl of fresh NBH with PTFE beads for serial amplification over a total of three rounds. Following each round of amplification, samples were evaluated by western blotting, as described below, for the presence of PrP^d^. For each sample, the number of positive rounds in each duplicate were tallied to allow for relative quantification of PrP^d^; thus, each sample could have a maximum score of 6 (e.g. three positive rounds in duplicate experiments), and a minimum score of zero. Scores were then normalized by dividing by the total number of rounds run (i.e. six for each sample) to arrive at a final score between 0-1. 

### Western blotting (WB) of sPMCAb amplified cerebrospinal fluid

 Following each round of amplification, an aliquot of each sonicated sample was subjected to western blotting for evaluation of PrP^d^ signal. Fifteen µl of sample homogenate were mixed with 7µl of sample buffer (0.1% [v/v] triton-X 100 and 4% [w/v] SDS in PBS), digested with 3µl proteinase-K at 500µg/ml (final concentration: 60µg/ml) for 20’ at 37°C followed by 10’ at 45°C. Twenty µl of this preparation were run on a pre-cast 12% bis-tris SDS-PAGE gel (Invitrogen) in a Bio-Rad electrophoresis apparatus for 1 hour at 160mV. Samples were then transferred to a PVDF membrane using a Transblot Turbo transfer system (Bio-Rad). PVDF membranes were subsequently probed with a PrP-specific monoclonal antibody (BAR224 conjugated to horse radish peroxidase, HRP) diluted 1:20,000 in 5% (w/v) powdered milk in 0.2% Tween-20 in tris-buffered saline (TBST) for 1 hour. Following washing with 0.5% Tween 20 in tris-buffered saline, immunoreactivity was detected using an enhanced chemiluminescent detection system (ECL-plus, Amersham Biosciences) in an LAS 3000 imaging system. (Fuji Photo Film, Fuji Inc, Valhalla, NY)

### Preparation of recombinant PrP for RT-QuIC

 Real-time quaking-induced conversion assays were performed with recombinant Syrian hamster PrP (SHrPrP) encoding residues 90-231 in pET41b and expressed and purified as previously described [39]. In brief, 1 liter cultures of lysogeny broth (LB) containing Auto Induction™ supplements (EMD Biosciences) were inoculated with SHrPrP-expressing Rosetta strain *E. coli*, grown overnight, and harvested when optical density (OD, 600 nm) of ~3 was reached. Cells were lysed with Bug Buster™ reagent with supplemented Lysonase™ (EMD Biosciences) and inclusion bodies (IB) were harvested by centrifugation of the lysate at 15,000 x g. IB pellets were washed twice and stored at -80 °C until purification (typically 24 hours or less). IB pellets were solubilized in 8 M guanidine hydrochloride (GuHCl) in 100 mM NaPO_4_ and 10 mM Tris pH 8.0, clarified by centrifugation at 15,000 g for 15’ and added to Super Flow nickel-nitrilotriacetic acid (Ni-NTA) resin (Qiagen) pre-equilibrated with denature buffer (6.0 M GuHCl, 100 mM NaPO_4_, 10 mM Tris pH 8.0). Denatured SHrPrP and Ni-NTA resin was incubated by rotating at room temperature for 45 min and then added to an XK fast protein liquid chromatography column (GE Healthcare). Refolding was achieved on column using a linear refolding gradient of denature buffer to refold buffer (100 mM NaPO_4_, 10 mM Tris pH 8.0) over 340 ml at 0.75 ml/min. SHrPrP was eluted with a linear gradient of refold buffer to elution buffer (100 mM NaPO4, 10 mM Tris pH 8.0 500 mM imidazole pH 5.5) over 100 ml at 2.0 ml/min. Fractions were pooled and dialyzed against two changes of 4 liters of dialysis buffer [20 mM NaPO_4_ pH 5.5). Recovered SHrPrP was adjusted to a final concentration of ~0.5 mg/ml.

### RT-QuIC analysis of cerebrospinal fluid

 Blinded cervid CSF samples were diluted 1:10 in RT-QuIC dilution buffer (PBS with 0.05% sodium dodecyl sulfate, SDS). Five µl of this 10^-1^ dilution were added to 95µl of RT-QuIC reaction buffer, consisting of 50mM NaPO_4_, 250mM NaCl, 1.0mM ethylenediaminetetraacetic acid tetrasodium salt (EDTA), 10µM Thioflavin T (ThT), and 0.1mg/ml Syrian hamster rPrP^C^, to yield a final volume of 100µl. Each test sample was repeated in triplicate in three separate experiments. Positive controls, consisting of 5µl of a 10^-3^ dilution of pooled CWD-positive brain from six experimentally infected white-tailed deer (cervid brain pool 6, CBP6) spiked into 95µl of RT-QuIC reaction buffer, were included in triplicate in each experiment. Negative controls, also repeated in triplicate, consisted of CSF samples from sham-inoculated white-tail deer prepared in a manner consistent with study deer, as well as untreated RT-QuIC reaction buffer spiked with 5µl of RT-QuIC dilution buffer. Reactions were prepared in a black 96-well, optical-bottom plate, which were then sealed and incubated in a BMG Labtech Polarstar^TM^ fluorimeter at 42°C for 48 hours (192 fifteen minute cycles) with intermittent shaking cycles; specifically 1 minute shakes (700rpm, double orbital pattern) interrupted by 1 minute rest periods. Thioflavin T fluorescence measurements (450nm excitation and 480nm emission) were taken every 15 minutes with the gain set at 1200, with the relative fluorescence units (RFU) for each triplicate sample progressively monitored against time with orbital averaging and 20 flashes/well at the 4mm setting. 

 Replicates were considered positive when they crossed a pre-defined positive threshold, calculated as five standard deviations above the mean fluorescence of all negative controls (CSF from sham-inoculated deer and untreated replicates spiked with RT-QuIC dilution buffer) in the assay. Times at which a sample crossed the positive threshold (C_t_) were recorded; samples remaining below the threshold were considered negative. C_t_ scores were derived by dividing the average C_t_ time of positive controls in an experiment by the average C_t_ times of individual triplicates from unknown CSF samples, resulting in RT-QuIC C_t_ scores typically between 0-1. Each sample was repeated in triplicate in three separate experiments to estimate the reproducibility of RT-QuIC scores. The fluorometric curves generated from sample amplification were analyzed using GraphPad Prism 5.0, with the real-time data from each well fit with a Boltzmann sigmoidal curve. From these curves, the slope and final (maximal) plateau fluorescence were determined. Parameters for each triplicate sample were averaged across the 3 experiments.

### Optimization of sPMCAb and RT-QuIC for use with cervid CSF

 To optimize each of the seeded amplification assays, we selected CSF from six study deer, inoculated with a variety of substrates through various routes, which were found to have large accumulations of PrP^d^ in their obex (i.e. immunohistochemical scores of 3/3 – deer 106, 110, 133, 144, 346, and 4116), and a sham inoculated deer (deer #4488) for dilutional analysis. For sPMCAb, we selected a range of spike volumes including 20μl, 10μl, and 1μl into 50μl of NBH. Samples were amplified as described above for sPMCAb, over a total of three rounds and evaluated by western blotting. For RT-QuIC, we selected a range of dilutions of CSF into RT-QuIC dilution buffer: 10° (i.e. no dilution), 10^-1^, and 10^-2^; 5μl of each dilution were spiked into 95μl of RT-QuIC amplification buffer and subjected to amplification over a 24hr period as described above.

### Immunohistochemistry of cervid obex

 Immunohistochemistry (IHC) was performed using the protocol described by Spraker et al [40]. Briefly, 3–5 mm sections of formalin fixed formic acid treated tissues were deparaffinized at 60–70°C for 1 hour, rehydrated via a series of xylene/ethanol baths, and treated in formic acid a second time (5 minutes) prior to a 20 minute antigen retrieval (Dako Target Retrieval Solution 10×) cycle in a 2100 Retriever™ (PickCell Laboratories). Slides were further processed with the aid of a Ventana Discovery™ autostainer utilizing the Ventana Red Map™ stain kit, the PrP^d^ specific primary antibody BAR224, and a biotinylated secondary goat anti mouse antibody (Ventana). After autostaining, the slides were quickly rinsed in a warm water detergent solution, passed through a series of graded alcohols and cover-slipped. Stained slides were blindly evaluated by two independent evaluators and assigned scores between 0-3, with zero being a complete absence of staining and three indicating intense PrP^d^-specific staining throughout the dorsal motor nucleus of the vagus and remainder of the obex. Scores were then averaged and divided by the maximum score (i.e. 3), to arrive at normalized IHC scores between 0-1. 

### Statistical analyses of experimental results

 To firstly evaluate if there was a relationship between sPMCAb, RT-QuIC, and IHC scores, we correlated normalized scores against one another using Spearman rank correlation. We then evaluated sPMCAb and RT-QuIC relative to IHC for sensitivity, specificity, positive and negative predictive values.

 We next evaluated and contrasted how well sPMCAb and RT-QuIC test scores were predicted by inoculum, genotype (GG/GS), month at which the individual was tonsil biopsy positive (by IHC), survival period (months), duration of clinical signs (weeks), and IHC obex scores. To undertake this we employed an Information Theoretic approach [41], whereby all single and pair-wise combinations of predictor variables were evaluated, using logistic regression, and ranked based on Akaike Information Criterion corrected for small sample size (AICc) [41]. This approach is of value because it enables determination of the most parsimonious model or set of models to explain sPMCAb and RT-QuIC scores, and also calculation of Variable Importance weights to determine the relative importance of one predictor variable over others [41]. Because Information Theory departs from frequentist based statistical approaches, which are dependent on *P*-values, we also calculated McFadden r^2^ values (appropriate for calculating fit of logistic regression models) so that the relative fit of models to the data could be assessed.

 The above analyses were also extended to evaluate how well predictor variables performed at predicting Boltzmann slope and maximum fluorescence for deer with positive RT-QuIC diagnoses. These values could only be assessed for deer returning a positive RT-QuIC result, causing the dataset to be reduced (n=14) and, accordingly, analyses were restricted to all pair-wise combinations of predictor variables so that the number of models did not greatly exceed the amount of data [41]. All analyses were conducted using the program R (www.r-project.org). Models were conducted based on a binomial data distribution, with the exception of models predicting maximum fluorescence which was based on a Gaussian distribution. Because models of maximum fluorescence were based on a Gaussian distribution, a regular coefficient of variation was computed, rather than McFadden r^2^.

## Results

### Immunohistochemical detection of PrP^d^ in cervid obex

**Figure 1:**
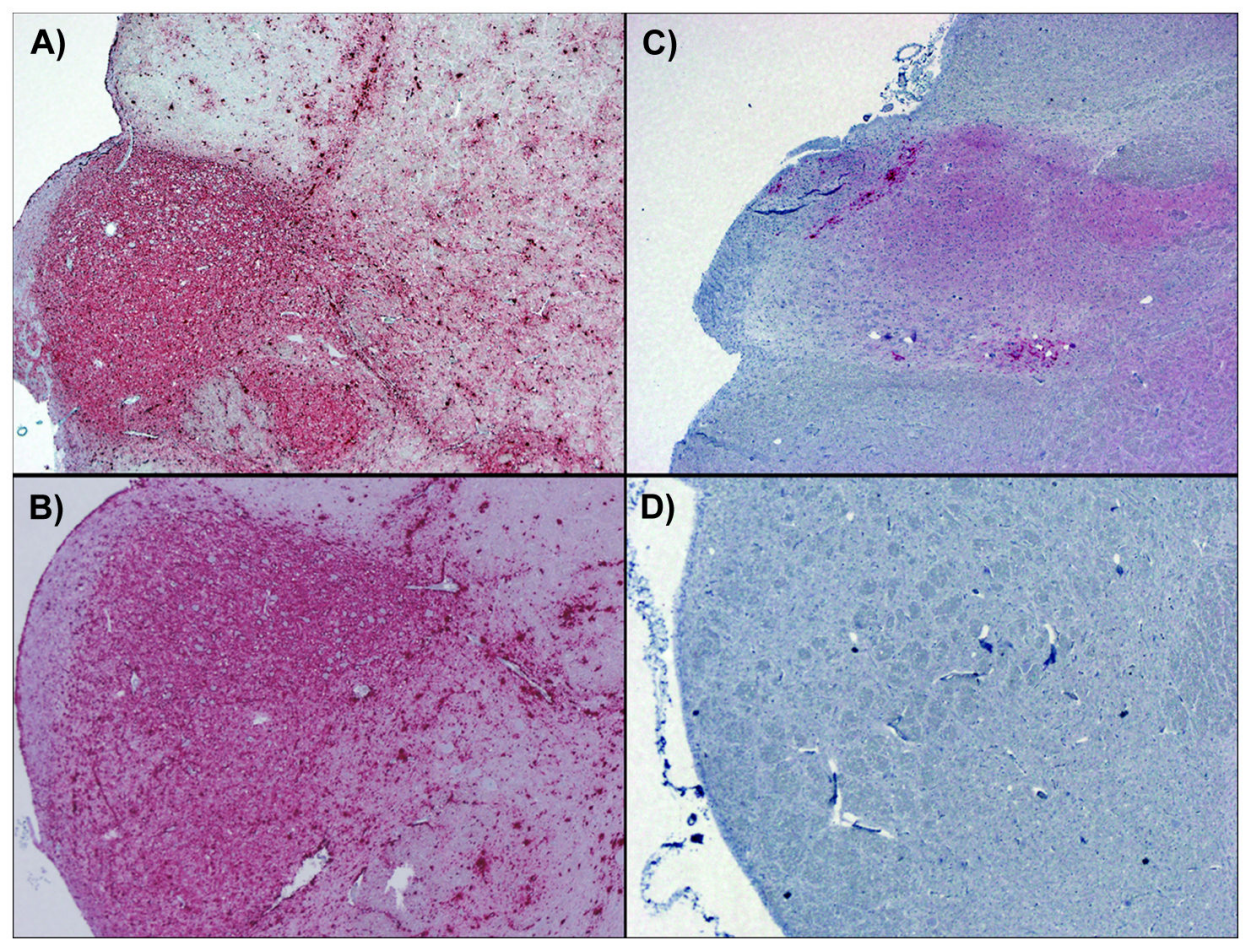
Immunohistochemistry of white-tailed deer obex. Immunohistochemistry results from deer #4502 (**A**), 4129 (**B**), 144 (**C**) and 4488 (**D**). Normalized scores from IHC+ animals ranged from 0.17 (e.g. #4129) to 1.0 (e.g. #4502 and 144). Sham-inoculated deer (e.g. #4488, D) were consistently negative by IHC.

 Using previously reported methods for IHC detection of PrP^d^ in cervid tissues, we analyzed obex collected at necropsy from each individual deer. Independent scores (0-3) from two separate evaluators were averaged and divided by the maximum score (i.e. 3), to arrive at a normalized score between 0-1. IHC demonstrated PrP^d^ in 26/48 individuals (Table 1), with normalized scores from positive animals ranging from 0.17-1 (mean: 0.77). (Figure **1**)

### Optimal spike volumes vary for sPMCAb and RT-QuIC

**Figure 2:**
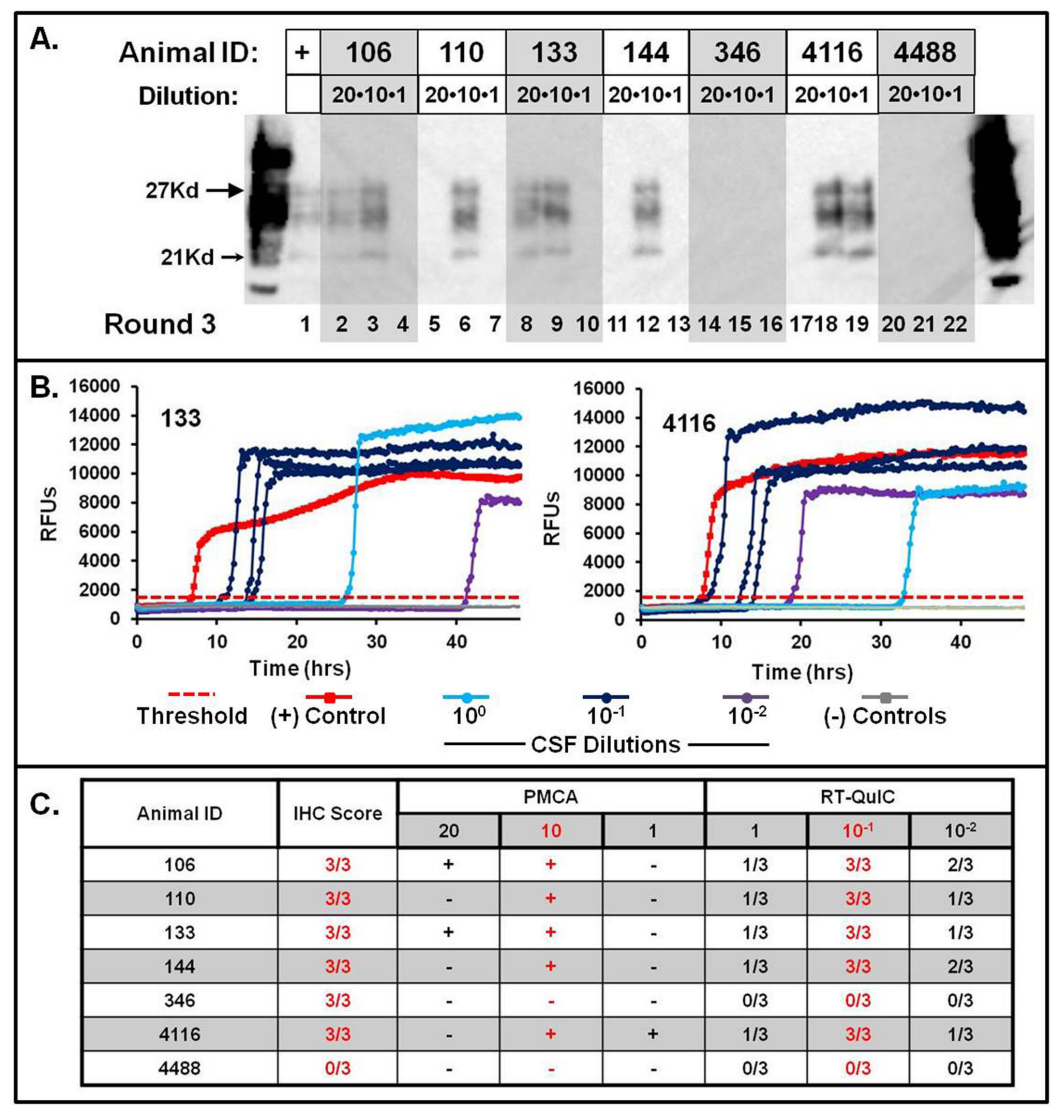
Optimization of sPMCAb and RT-QuIC. Various spike volumes and dilutions were chosen for optimization of each assay for use with cervid CSF. Cerebrospinal fluid from six CWD-positive deer (#106, 110, 133, 144, 346, and 4116) and one CWD-negative deer (#4488) were chosen for optimization. With sPMCAb (A.), spike volumes including 20μl (Lanes 2, 5, 8, 11, 14, 17, and 20), 10μl (Lanes 3, 6, 9, 12, 15 18, and 21), and 1μl (Lanes 4, 7, 10, 13, 16, 19, and 22) were evaluated using three rounds of sPMCAb. For RT-QuIC (B.), dilutions of CSF including 10°, 10^-1^, and 10^-2^ were evaluated in 48hr experiments. The threshold for positive amplification (C_t_) is represented by the horizontal hashed red line. Data encompassing results from IHC, sPMCAb, and RT-QuIC from CSF samples from seven deer (C.) were evaluated to estimate the optimum spike volumes and dilutions of CSF, and revealed that 10μl of whole CSF appeared to be optimal for sPMCAb, while a 10^-1^ dilution of CSF had the greatest precision in RT-QuIC analysis.

 To evaluate the optimal volumes for seeded amplification in both sPMCAb and RT-QuIC, we evaluated a range of starting volumes and dilutions of CSF from six CWD-positive deer in each assay: 20μl, 10μl and 1μl of CSF into 50μl of NBH with sPMCAb, and 10°, 10^-1^, and 10^-2^ dilutions of CSF in RT-QuIC. For sPMCAb, we observed the most efficient seeded amplification with 10μl of spike volume (5/6 showing positive amplification). Less efficient amplification was observed with 20μl (2/6 positive) and 1μl (1/6 positive) spike volumes. For RT-QuIC, seeded amplification had the highest precision when using 10^-1^ dilutions of CSF, showing seeded amplification in 3/3 replicates in 5/6 CSF samples; higher and lower dilutions of CSF showed less repeatable amplification. (Figure **2**)

### Serial PMCA prion seeding activity in cerebrospinal fluid of white-tailed deer

**Figure 3:**
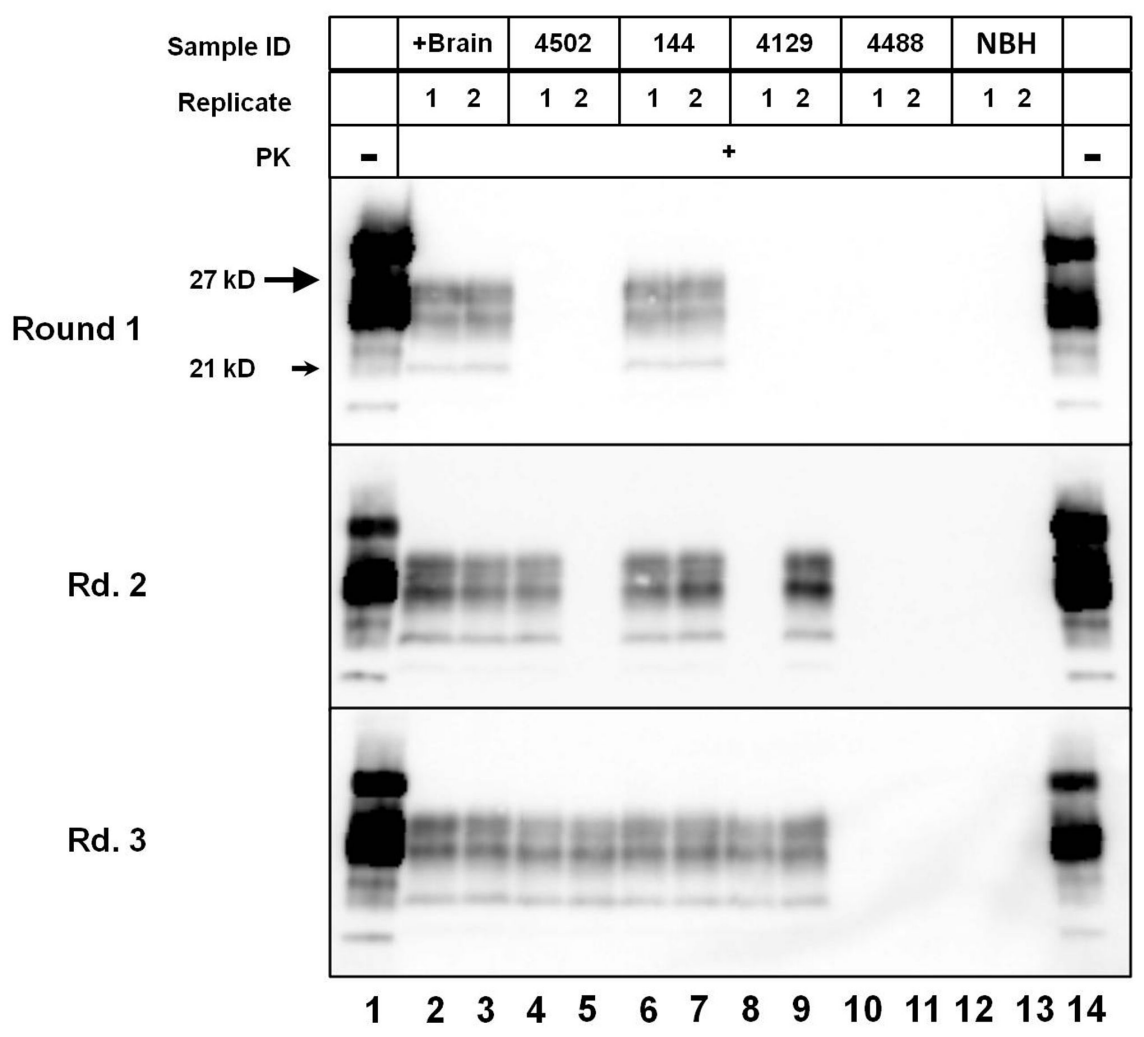
Serial PMCAb detection of prion seeding activity in CSF from CWD-positive deer. Cerebrospinal fluid samples, along with positive and negative controls, were assayed in duplicate for three rounds of sPMCAb, with the total number of positive rounds tallied for each sample. Positive control samples were positive in each duplicate through all rounds (Lanes 2 and 3, for a total of 6/6 positive rounds and a normalized score of 1), while CSF samples varied in their total number of positive rounds. CSF from deer #4502 (Lanes 4 and 5) and #4129 (Lanes 8 and 9) demonstrated amplification in 3/6 rounds of sPMCAb, for a normalized score of 0.5, while CSF from deer 144 was positive in 6/6 rounds (Lanes 6 and 7). Negative controls, including deer #4488 (Lanes 11 and 12) and unspiked brain homogenate (NBH – lanes 12 and 13) remained negative throughout all rounds.

Using a modified sPMCA technique incorporating PTFE beads (sPMCAb) [30], amplification was observed in 16/48 cerebrospinal fluid samples (Table 1). The mean number of rounds positive in amplified samples was 5.1 out of 6, ranging from 0-3 rounds for each duplicate and yielding sPMCAb scores from 0.17-1.0 (mean: 0.85). A single aliquot (i.e. 1/56 replicates from CWD-negative control samples) of CSF from a sham-inoculated deer (deer 123) yielded a false positive result in a single replicate in sPMCAb, though failed to demonstrate amplification in RT-QuIC (see below). The remaining controls (CSF from additional negative control deer, additional aliquots of CSF from deer 123, and unspiked negative controls) failed to amplify PrP^d^ during the course of sPMCAb analysis. (Figure **3**)

### RT-QuIC detects prion seeding activity in cerebrospinal fluid of white-tailed deer

**Figure 4:**
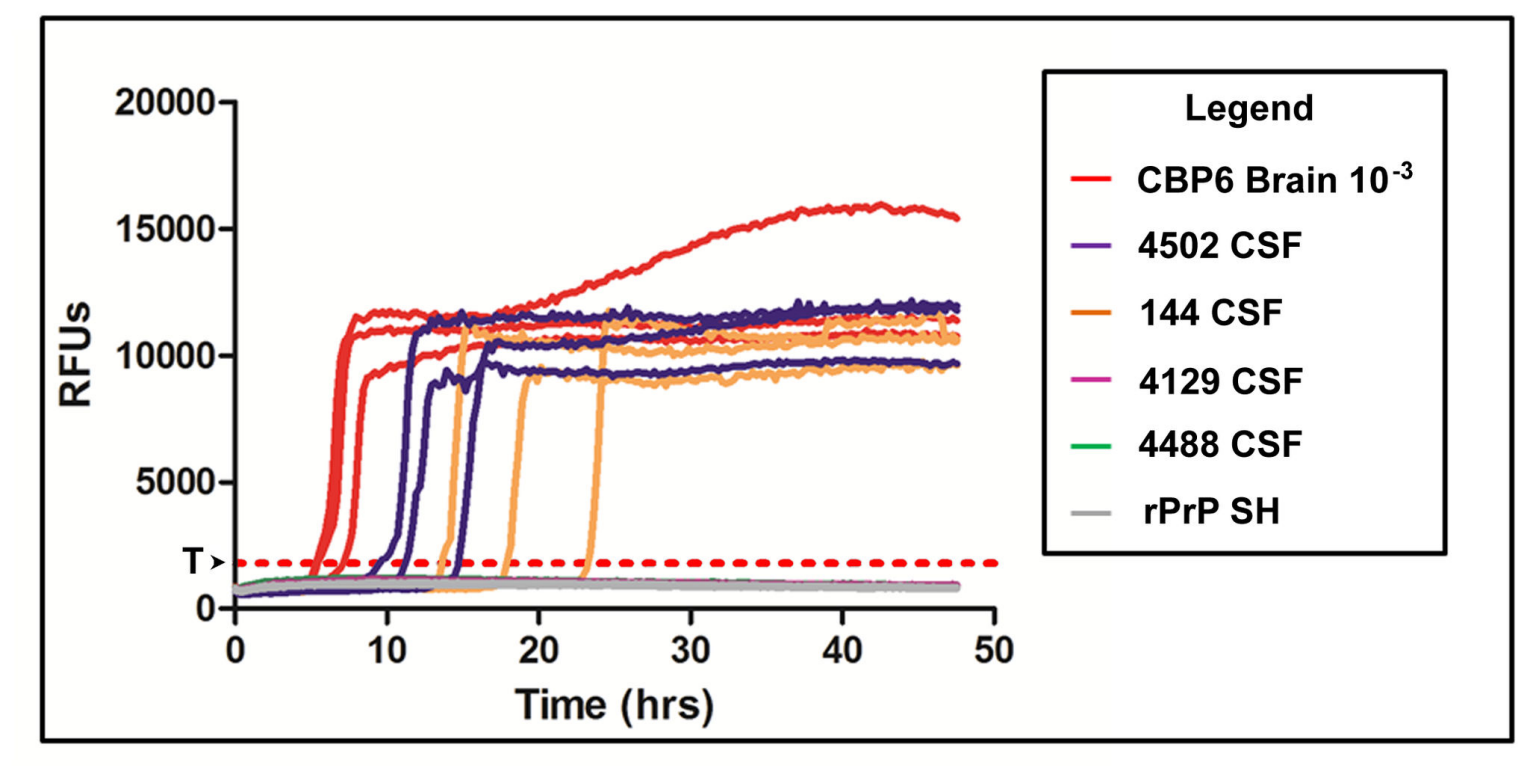
RT-QuIC seeded amplification detected by ThT fluorescence in CSF of deer. A 10^-3^ dilution of cervid brain pool (red), as well as 10^-1^ dilutions of CSF from deer #4502 (blue) and 144 (orange) showed evidence of prion seeding activity (ThT binding) in 48 hour RT-QuIC experiments. The time to reaching threshold fluorescence (C_t_ – red horizontal dashed line) varied amongst RT-QuIC positive animals, and correlated with IHC scores. CSF from some CWD-positive deer (e.g. deer #4129), all sham-inoculated deer (e.g. deer #4488), and all untreated controls failed demonstrate seeding activity.

An RT-QuIC assay employing truncated Syrian hamster recombinant prion protein [13] as a substrate successfully amplified CWD prion seeding activity in 14/48 CSF samples (Table 1). By comparing threshold times to those of a 10^-3^ dilution of CBP6 positive control, resultant RT-QuIC scores from positive animals ranged from 0.2-1.0, with a mean score of 0.47, inferring that the level of prion-seeding activity was multiple orders of magnitude lower than that found in the CNS. All samples were repeated in three separate experiments, and negative controls (CSF from sham-inoculated deer and untreated controls spiked with RT-QuIC dilution buffer) remained negative throughout the course of analysis. Over the course of multiple experiments, we found that RT-QuIC scores were fairly reproducible, with standard error of C_t_ scores between separate experiments ranging from 0.01-0.07. (Figure **4**)

### Inter-assay correlation and relationship to available historical data

 All three assays (IHC, sPMCAb, and RT-QuIC) were positively correlated (Table **2**). The strength of agreement was greatest between sPMCAb and RT-QuIC and less strong vs. IHC. Prion seeding activity in the CSF was detected by sPMCAb and RT-QuIC in ~50% of obex IHC-positive deer (Table **3**). Both amplification assays were more likely to align with IHC-negative results (specificity), and deer with a positive sPMCAb or RT-QuIC score had a high probability of detection by IHC (positive predictive value). However, absence of amplification by sPMCAb or RT-QuIC was only moderately likely to align with absence of detection by IHC (negative predictive value). Overall, sPMCAb and RT-QuIC were broadly comparable.

**Table 2:** Spearman correlation of diagnostic methods.

	sPMCAb	IHC	RT-QuIC
sPMCAb		0.593	0.778
IHC	<0.001		0.690
RT-QuIC	<0.001	<0.001	

Upper diagonal represents correlation coefficients (*ρ*) and lower diagonal corresponding *P*-values.

**Table 3:** Evaluation of how PMCA and QuIC compare to IHC in identification of CWD-positive deer.

	sPMCAb	RT-QuIC
Sensitivity	53.8	50.0
Specificity	90.9	95.5
Positive predictive value	87.5	92.9
Negative predictive value	62.5	61.8

**Figure 5:**
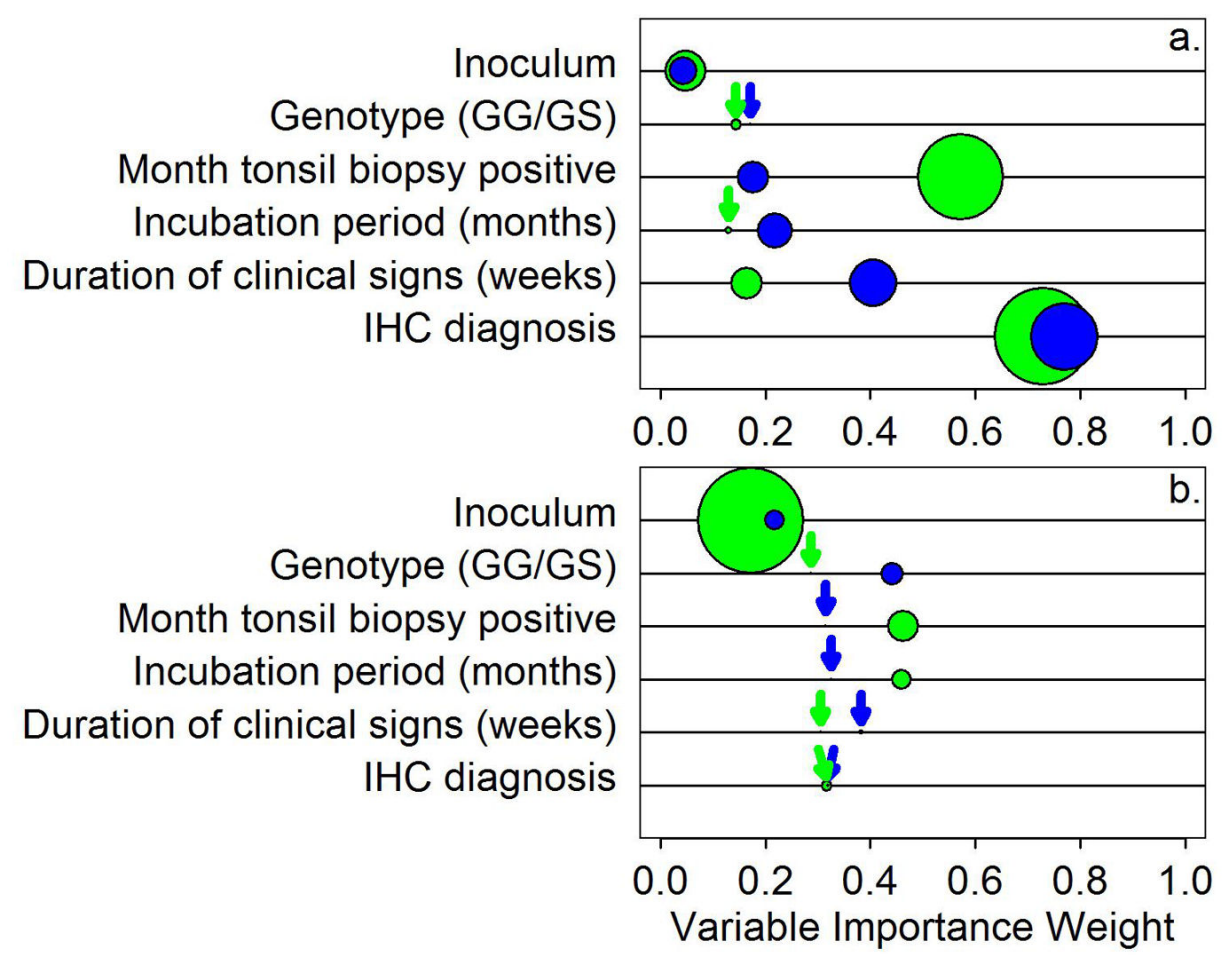
The relative importance of predictor variables for (a) sPMCAb (blue circles) and RT-QuIC (green circles) scores, and (b) Boltzmann slope (blue circles) and maximum fluorescence (green circles). Size of circles represents McFadden r^2^ values (range <0.001 – 0.292). Where r^2^ values were <0.03 a blue or green arrow indicates their position for clarity. Although inoculum r^2^ values were often relatively high (larger circles) compared to other predictors, their importance was low indicating that the high r^2^ values for this variable was a statistical artifact of the high number of inoculum categories.

Using regressions and Information Theory, we found that sPMCAb and RT-QuIC diagnostic scores were predicted by single and pair-wise combinations of inoculum, genotype, month tonsil biopsy positive, incubation period, duration of clinical signs and IHC diagnosis (Table **S2**). IHC-positivity was the best (most parsimonious) predictor of sPMCAb-positivity (Figure 4a, slope coefficient ± SE 2.371 ± 1.108). Next in variable importance weight was duration of clinical signs (slope coefficient ± SE 0.112 ± 0.065), while the remaining predictors were less important. IHC score was also the best predictor of RT-QuIC-positivity (Figure 4, slope coefficient ± SE 4.190 ± 2.576). The month at which an animal was tonsil biopsy positive was the second best predictor (slope coefficient ± SE -0.212 ± 0.117), inferring that a longer period for which prions might accumulate in the CNS favored prion entry into the CSF. For both sPMCAb and RT-QuIC, McFadden r^2^ values were generally related to variable importance weights (Figure **4a**), except for inoculum. However, inoculum source and type was a relatively unimportant predictor variable, owing to the number of categories within this predictor causing low model parsimony (Figure **5a**, Table **S2**). Boltzmann slope, a modeled, best fit slope based on available data, for RT-QuIC yielded low McFadden r^2^ values (Figure **5b**, Tables **S1** and **S3**). A similar pattern was observed for predictors of Boltzmann maximum fluorescence for RT-QuIC, although inoculum was distinctly less important than the other variables (Figure **5b**, Tables **S1** and **S3**). 

## Discussion

As the only known prion disease affecting free-ranging animals, the early and antemortem detection of chronic wasting disease infection in cervids is of paramount importance in disease management. The detection of CWD and other prions in bodily fluids, including saliva and CSF, has been the subject of recent research [26,42]. In the present study, we compared sPMCAb and RT-QuIC analysis of CSF from CWD-exposed cervids in relation to IHC detection of PrP^d^ in the obex region of the brain and other available historical information (e.g. inoculum source, incubation period, genotype, etc.). 

 We found that results from both *in vitro* detection assays (sPMCAb and RT-QuIC) were highly correlated, with ~50% of deer with IHC-positive PrP^d^ in the obex showing demonstrable prion seeding activity in the CSF. Assay agreement was fairly high between sPMCAb and RT-QuIC, and while sPMCAb identified slightly more IHC-positive animals than RT-QuIC (16/26 vs. 14/26 for RT-QuIC), assay scores seemed to correlate slightly better between RT-QuIC and IHC using Spearman correlation (0.690 vs. 0.593 for sPMCAb). These results are consistent with a previous study evaluating the CSF of CWD-infected Rocky Mountain elk (*Cervus elaphus nelsoni*), in which elk with higher levels of PrP^d^ demonstrated by IHC of the obex were more likely to be sPMCA positive [26]. In addition to our findings in samples from CWD-positive animals, sPMCAb produced a false positive result from a single aliquot of CSF from a negative control deer (1/56 total replicates), implying slight deficits in specificity for this assay. Previous experiments in our lab and elsewhere have found specificities of <100% for three-round sPMCA [36], so these results perhaps are not surprising. As amplification was only observed in a single aliquot of CSF from one of the negative control deer, while additional aliquots from the same deer remained negative, sample contamination seems probable.

A number of previous studies employing amplification assays for the detection of PrP^d^ in CSF samples have been reported, predominantly in IHC-confirmed cases of CJD in humans and scrapie of hamsters [27,28]. In these reports, seeded amplification was observed in 75-80% of CJD or scrapie-positive individuals. Another study evaluating the CSF of Rocky Mountain elk by sPMCA was only able amplify protease-resistant prion from 3/15 (20%) samples from CWD-positive animals, somewhat lower than the percentage of prion-seeding activity seen in the CSF of white-tailed deer in the present study. Taken collectively, this seems to suggest that prionorrhachia (shedding of prions into the CSF) is either intermittent or inconsistent in CWD-infected cervids, or levels of prion seeding activity are very low, compared to scrapie-infected hamsters or CJD-infected humans. A systematic evaluation of TSE-infected CNS tissues of deer and other species may help pinpoint the probable source of CSF shedding (e.g. ependymal cells [43]) via correlating histopathological or PrP^d^-deposition analysis to amplification results. 

In addition to comparing inter-assay results, we sought correlations between amplification results and other available historical data from experimental animals, a study component our laboratory is uniquely suited to undertake. We found that two aspects of the clinical history – duration of clinical signs and earliest time point of tonsil biopsy positivity – were positively correlated to sPMCAb and RT-QuIC results, respectively. This finding seems to imply that the longitudinal accumulation of CWD prions in the central nervous system of deer, either following extended incubation periods or early access to the lymphoreticular system – the predominant amplification site in affected deer – has an important impact on the accumulation of detectable prions in the cerebrospinal fluid. In previous studies, we have found a correlation between genotype and successful amplification of CWD prions in lymphoreticular tissues [11]; this finding does not appear to translate to seeded amplification in the CSF of deer as there was little correlation between genotype and likelihood of detection via either sPMCAb or RT-QuIC. 

 Apart from simply investigating the presence or absence of seeding activity, we also examined relationships between available historical data and advanced analysis of RT-QuIC amplification curves, to determine whether additional insights could be gained into the relationships of seeding activity curves and sample source. We focused on amplification amplitude and slope, and attempted to correlate these to each of the *a priori* variables. In this examination, we found no substantive relationship of any of the variables to either mean slope or peak fluorescence. These results may imply that these metrics of RT-QuIC analysis do not arise from inherent properties of the amplifiable prions present in the CSF, or may be due to the small sample size available for the statistical analyses. We are presently analyzing additional samples from multiple cervid sources to further examine whether advanced amplification metrics may correlate to certain aspects of an individual’s clinical history. 

In summary, the present study evaluated two amplification assays – sPMCAb and RT-QuIC – for their ability to amplify PrP^d^ in the CSF of CWD-exposed and naïve white-tailed deer. Results between the two assays correlated well with each other and to IHC results from obex collected at necropsy, albeit with reduced sensitivity. *A priori* variables, notably date of tonsil biopsy positivity and duration of clinical signs, influenced the likelihood of a sample being positive by either assay, while our post-amplification analyses (slope of curve or peak fluorescence) did not correlate with clinical histories. Based on our findings, we believe that amplification assays hold continued promise in the detection of prion-infection using post- or ante-mortem samples and our future work will continue to evaluate the utility of these assays in detecting seeding activity in these tissues and biological fluids. 

## Supporting Information

Table S1 **Summary of genotype, incubation period and advanced RT-QuIC metrics from study deer.** Neither genotype nor incubation period were significantly correlated to sPMCA or RT-QuIC results; while advanced metrics derived from RT-QuIC experiments had no direct correlation with any of the historical variables from CWD-exposed or sham inoculated deer.
(XLSX)

Table S2 **Comparison of predictor models to estimate sPMCAb and RT-QuIC scores.** Models represent logistic regressions of all individual and pair-wise combinations of predictors, and are ranked from most parsimonious to least parsimonious by ∆AICc. 1=Inoculum (blood and blood products, environment, urine/feces, negative, brain, saliva), 2=IHC, 3=Genotype (GG/GS), 4=Month sample was tonsil biopsy positive (by IHC), 5=Incubation period (months), and 6=Duration of clinical signs (weeks). K=number of model parameters, *w*=model weight, r^2^=McFadden’s r^2^.
(XLSX)

Table S3 **Comparison of predictor models to estimate Boltzmann slope and maximum fluorescence of samples diagnosed as CDW positive by QuIC.** Models represent logistic regressions for slope and linear regressions for fluorescence of all pair-wise combinations of predictors, and are ranked from most parsimonious to least parsimonious by ∆AICc. 1=Inoculum (blood and blood products, environment, urine/feces, negative, brain, saliva), 2=IHC, 3=Genotype (GG/GS), 4=Month sample was tonsil biopsy positive (by IHC), 5=Incubation period (months), and 6=Duration of clinical signs (weeks). K=number of model parameters, *w*=model weight, r^2^=McFadden’s r^2^ for slope and r^2^ for fluorescence.
(XLSX)
